# Scoring-Rule Dependence and Sign Instability in Vision–Language Covariate-Shift Monitoring for Camera Sensors

**DOI:** 10.3390/s26206432

**Published:** 2026-10-12

**Authors:** Loránt Szabó, Zoltán Weltsch

**Affiliations:** 1 AI Research Center, John von Neumann University, 6000 Kecskemét, Hungary; 2 Department of Road and Rail Vehicles, Széchenyi István University, 9026 Győr, Hungary; weltsch.zoltan@sze.hu

**Keywords:** covariate shift, camera sensors, out-of-distribution detection, CLIP, vision–language models, score calibration, prompt sensitivity, sensor monitoring

## Abstract

Camera-distribution monitoring and semantic out-of-distribution detection assign different meanings to weather and illumination changes. We evaluate specified departures from a camera reference distribution and distinguish the image representation from its scoring rule. Frozen CLIP ViT-B/32 gives AUROC 0.504 on a fixed PUG covariate-shift sample with content-prompt maximum cosine, but an ID-fitted Mahalanobis readout of the same encoder reaches 0.809. On BDD100K night, a text-free CLIP KNN readout reaches 0.913 versus 0.534 for maximum cosine. Prompt/scoring controls further demonstrate readout dependence and the effect of named-condition prior knowledge. On Boreas, the baseline ranks winter and evening shifts in opposite directions (AUROC 0.823 and 0.249). One threshold fitted on 500 separate summer calibration frames misses 44.8% of winter and 99.6% of evening frames, with 5.8% false alarms on held-out summer frames. Scene and position controls partly address acquisition confounding. A preprocessing audit overturns the original adversarial-training benefit; corrected checkpoint results and a separate five-seed study replace that conclusion. These results support evaluating complete encoder–score–prompt–calibration pipelines, with explicit sampling and operational limits.

## 1. Introduction

A perception system may encounter a new semantic category, a changed imaging condition, or both. These events require an explicit operational definition. A semantic OOD detector may appropriately accept familiar objects photographed in rain, whereas a camera-distribution monitor may flag rain as a departure from its commissioning distribution. Neither an alarm nor acceptance alone establishes whether the downstream perception system is correct or safe.

Vision–language models such as CLIP [1] support several OOD scores, including maximum concept matching (MCM) [2] and negative-label methods [3]. The image encoder, text bank, scoring map and calibration procedure jointly define the detector. A score can discard information present in its input embedding. Consequently, an AUROC near 0.5 for maximum text–image cosine cannot establish that the underlying representation lacks information about a shift.

This study examines that distinction using synthetic factor changes in PUG [4], matched clean/corrupted images from CIFAR-10-C [5], and real driving populations from BDD100K [6] and Boreas [7]. The original content-prompt score has weak discrimination on several populations and reverses its ranking direction between winter and evening on Boreas. Additional readouts of the same frozen CLIP encoder provide direct counterexamples to a representation-wide explanation. The resulting question is which part of the evaluated pipeline supports a given monitoring claim.

**Figure 1:**
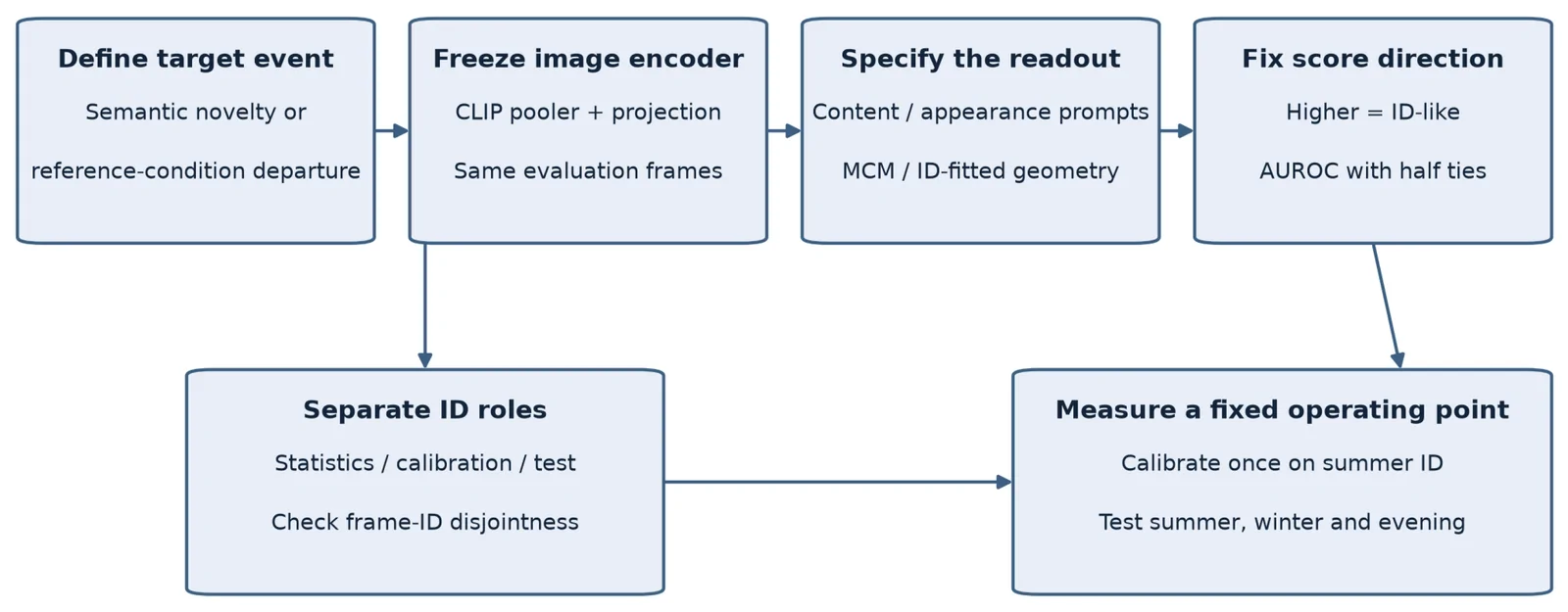
Study design and information flow. Encoder fitting, ID statistics, prompt/scoring choices, and threshold calibration have distinct roles. The separate calibration experiment retains one threshold across winter and evening.

The contributions are (i) a same-encoder comparison of text-free Mahalanobis/KNN and content-prompt scores before and after visual projection; (ii) explicit prompt and scoring controls, with their different information requirements disclosed; (iii) a fixed, separately ID-calibrated operating-point analysis across Boreas conditions and partial controls for scene/position confounding; and (iv) an auditable account of score direction, source-version differences, and remaining sampling limitations. Existing sensor-chain, camera–LiDAR, and embedded measurements are retained with narrower interpretations. A preprocessing audit corrects the adversarial-training and CIFAR-10-C comparisons, with a separate five-seed training sensitivity study. No detector-aware attack result is claimed in this manuscript. Figure 1 shows how the encoder, the ID statistics, the prompt and score choice, and the threshold calibration enter separately, and which of them each reported result is a statement about.

### Relation to Prior Work and Task Scope

Full-spectrum OOD detection explicitly tolerates covariate-shifted ID while detecting semantic novelty [8], and the benchmark line built on that decomposition—OpenOOD [9] and its v1.5 revision, which extends it to foundation backbones including CLIP [10], dedicated full-spectrum methods [11], and the broader taxonomy [12]—treats appearance change as something to be tolerated rather than reported. The weather/time-of-day labels below instead define a *distribution-monitoring* target: clear daytime or summer frames are the reference, and selected alternative conditions are monitored departures. Failure to alarm on these populations is therefore a failure relative to this chosen target, not a contradiction of the full-spectrum objective. Conversely, successful weather detection does not demonstrate unseen-class detection. That the two axes interact rather than decompose cleanly is itself established: detector behavior depends on how near the shift is to the reference distribution [13], and appearance robustness and OOD behavior do not vary together across corruption families [14]. Two results from the full-spectrum line motivate keeping the axes apart by construction: apparent detector gains can be driven by covariate rather than semantic shift [15], and whether an input counts as OOD at all is a property of the model and of the task asked of it rather than of the data alone [16].

For vision–language detectors specifically, surveys of the CLIP-based OOD literature [17,18] and a real-world benchmark reporting that CLIP-based detectors struggle to varying degrees on realistic shifts [19] report a wide spread of outcomes across scoring rules and benchmarks. The present results are consistent with that spread and locate part of its cause: as Section 3.2 shows, the same frozen encoder moves from chance to well above chance when only the read-out is changed, so a result reported for “CLIP” is a statement about an encoder–score–prompt–calibration pipeline and not about the encoder. Safety-monitoring surveys for learned perception [20] and analyses of data shift in automotive object detection [21] frame the monitoring target used here.

MSP [22], Energy [23], Mahalanobis [24] and KNN [25] provide different summaries of a pretrained network. MCM normalizes concept similarities across an ID label bank [2], whereas maximum cosine does not. NegLabel introduces a negative-label bank [3], and zero-shot outlier exposure synthesizes candidate outlier descriptions [26]. We evaluate specified instances of these ideas rather than the entire family of VLM-based detectors, and the two we implement are adaptations to this monitoring target rather than reproductions of their original ImageNet-scale protocols. CLIP/SigLIP comparisons [27] are between released checkpoints and do not isolate the training loss.

## 2. Methods and Evaluation Design

### 2.1. Score Direction, Discrimination, and Operating Points

All reported scores are oriented before evaluation so that higher means more ID-like. For independent draws from the specified ID and monitored populations, the discrimination estimand is

(1) $$A=\mathrm{Pr}\left\{s\left(X_{\mathrm{ID}}\right)>s\left(X_{\mathrm{shift}}\right)\right\}+\frac{1}{2}\mathrm{Pr}\left\{s\left(X_{\mathrm{ID}}\right)=s\left(X_{\mathrm{shift}}\right)\right\}.$$

The empirical AUROC estimates this pair-ranking probability; ties receive half weight. Scores below a threshold trigger an alarm: a(x)=1{s(x)<τ}; equality is retained. The ID false-alarm rate is ${\mathrm{Pr}}_{\mathrm{ID}}\left(s<\tau \right)$ and the shift miss rate is ${\mathrm{Pr}}_{\mathrm{shift}}\left(s\geq \tau \right)$. An AUROC below 0.5 describes reversed ranking under this fixed convention; it does not specify either operating rate.

For benchmark FPR95, ID is the positive class: it is the fraction of shifted/OOD examples accepted at an empirical threshold retaining at least 95% of the ID evaluation sample. It is thus an OOD *miss* rate at that benchmark operating point, not a 95% OOD-detection requirement and not the ID false-alarm rate. The separate-calibration experiment in Section 4.2 uses a different ID sample for threshold selection and reports the resulting held-out rates. No score is flipped after observing test AUROC.

### 2.2. Scoring Functions

For classifier logits $z_{k}$, we use $s_{\mathrm{MSP}}=\max_{k}\mathrm{softmax}{\left(z\right)}_{k}$ and $s_{\mathrm{Energy}}=\log\sum_{k}\exp\left(z_{k}\right)$, the negative of the conventional energy, to keep the ID-high convention. A class-conditional Mahalanobis score is

(2) $$s_{M}\left(x\right)=-\min_{c}{\left(f\left(x\right)-{\hat{\mu }}_{c}\right)}^{⊤}{\hat{\Sigma }}^{-1}\left(f\left(x\right)-{\hat{\mu }}_{c}\right).$$

An unconditional readout uses one mean. Logit-calibration scores such as ODIN [28] and statistical multi-layer summaries [29] are further members of this family that are not evaluated here. The real-data ResNet50 [30] baseline uses ImageNet-pretrained IMAGENET1K_V2 weights without image-encoder fine-tuning on either driving dataset. Its BDD means are fitted by scene category on 3000 clear-daytime training frames with tied within-scene covariance plus $10^{-3}I$; Boreas uses 500 summer statistics frames and one Gaussian. These detectors use ID data for fitting and are not wholly zero-shot pipelines.

Let h(x) be CLIP’s unnormalized 768-dimensional visual pooler output, $v\left(x\right)=Wh\left(x\right)/{∥Wh\left(x\right)∥}_{2}$ its 512-dimensional projected embedding, and $t_{c}$ a normalized text embedding. The baseline is raw maximum cosine,

(3) $$s_{\max}\left(x\right)=\max_{c\in C}v{\left(x\right)}^{⊤}t_{c}.$$

No learned temperature is applied to this baseline. In the text-free control, unconditional Mahalanobis is fitted separately in *h* and *v*, with $\hat{\Sigma }=n^{-1}\sum_{i}\left(f_{i}-\hat{\mu }\right){\left(f_{i}-\hat{\mu }\right)}^{⊤}+10^{-3}I$ in float64. KNN first L2-normalizes features in either space and returns the negative cosine distance to the 50th nearest ID statistics vector. The encoder stays frozen; the fitted ID distribution is additional information unavailable to the text-only baseline. Differences between the two feature spaces also involve the dimensionality, normalization, and regularization scales, so they do not isolate a causal effect of projection.

The archived Fisher score combines exactly MSP, Energy, and one ResNet Mahalanobis score; CLIP is not a constituent. For ID calibration size *n*, let $p_{j}\left(x\right)=\max\left\{1/\left(n+1\right),{\hat{F}}_{j,n}\left(s_{j}\left(x\right)\right)\right\}$, where the right-continuous ECDF counts calibration scores at or below $s_{j}\left(x\right)$. The reported ID-high score is $s_{F}=2\sum_{j=1}^{3}\log p_{j}$, the negative of the usual Fisher combination statistic [31]. This is an empirical ranking baseline. The components are dependent, and the clipped ECDF values do not justify an automatic $\chi^{2}$ null law, finite-sample error guarantee or a guarantee that the fusion matches its best constituent. Fisher *p*-value combination is unrelated to Fisher information.

### 2.3. Populations, Splits, and Models

PUG permits rendered category and appearance factors to be specified separately. The replayed CLIP protocol takes the first 50 animal identities and first 30 environments in metadata encounter order as ID support. ID also requires the Default texture. CS retains ID animals with either an environment outside that support or a nondefault texture. SS uses held-out animals in ID environments with default texture. Thus, CS is not solely an environment intervention. Seed 42 defines the fixed draw: 500 ID images are reserved for statistics and 1000 ID evaluation images are sampled from the remainder; CS and SS use 1000 images each. Cached frame IDs verify statistics/evaluation disjointness. These are sample-level measurements, not an evaluation of the full PUG corpus or repeated data-split seeds. The exact animal template is “A photo of a {class} in a natural environment”, rather than the shorter template stated in the original draft.

The real-data populations are listed in Table 1. BDD’s night subset also requires clear weather, rain/snow subsets require daytime, and fog pools require available times of day. Attribute selection defines observational strata, not isolated causal weather changes. The populations can differ in scene, geography, traffic, and camera source. Boreas uses a repeated platform/route, but the original 500-frame seasonal samples are time-sampled rather than position-paired. Separate statistics, ECDF-calibration, and ID evaluation frame lists are retained unchanged in Supplementary Materials File S1. Their frame identifiers are disjoint, although adjacent frames and repeated route segments can remain dependent.

CLIP controls use openai/clip-vit-base-patch32. The driving content bank is “a photo of a {scene} scene” for city street, highway, residential, parking lot, tunnel, and gas stations, on both BDD and Boreas. The PUG checkpoint comparison additionally uses openai/clip-vit-base-patch16 and google/siglip-base-patch16-224. The original detection runs used PyTorch 2.10/MPS on an Apple M4 Pro (Apple Inc., Cupertino, CA, USA); the additional frozen-CLIP controls record Python 3.13.5, PyTorch 2.10.0, Transformers 5.2.0, and NumPy 2.3.5 in File S6. Historical runs used a different Transformer version, so exact run provenance is retained rather than assuming an identical software stack. The new control baseline reproduces all eight rounded PUG/driving CLIP cells; CPU recalculation from cached embeddings reproduces all 32 text-free AUROCs within $5.1\times 10^{-7}$ of their archived six-decimal values.

### 2.4. Prompt and Alternative-Score Controls

On the same cached driving-image embeddings, five configurations are evaluated: the six content prompts; five appearance prompts; their 30 scene–condition compositions; five unrelated-object scene prompts; and a signed contrast using the five appearance prompts. The appearance strings are “a photo taken on a clear day”, “a photo taken at night”, “a photo taken in heavy rain”, “a photo taken in snow”, and “a photo taken in fog”. The contrast is

(4) $$s_{\mathrm{app}}\left(x\right)=v{\left(x\right)}^{⊤}t_{\mathrm{clear}}-\max_{c\in \{\mathrm{night},\mathrm{rain},\mathrm{snow},\mathrm{fog}\}}v{\left(x\right)}^{⊤}t_{c}.$$

Its direction follows the designated clear-day reference. It uses prior knowledge of the named monitored conditions, with no per-image condition label supplied at inference. Because the prompts were specified for these study conditions, the control is exploratory and shift-informed. It does not establish performance on unanticipated shifts. The contrast changes the scoring map as well as the prompt content. Prompt count and token length are not fully matched across banks; the full strings are in File S6.

MCM [2] uses $s_{\mathrm{MCM},T}\left(x\right)=\max_{c}\exp\left(v^{⊤}t_{c}/T\right)/\sum_{j}\exp\left(v^{⊤}t_{j}/T\right)$. We report T∈{0.01,0.1,1} without selecting a winner on test labels; T=1 is the source method’s default. A NegLabel-inspired adaptation [3] uses the ID similarity mass divided by ID-plus-negative mass, with 10,000 negative labels and T=0.01. This implementation uses filtered WordNet nouns only, ranks candidates by $1-\max_{c}\cos\left(t_{\mathrm{neg}},t_{c}\right)$, and uses one ungrouped bank. The candidate pool is every lemma name of every noun synset returned by the NLTK WordNet corpus reader, with underscores replaced by spaces, deduplicated and sorted, and then filtered to ASCII strings of 2–30 characters; this leaves 118,302 candidates. That count is the figure a reader should reproduce before regenerating the bank because the provenance record fixes the corpus as NLTK WordNet noun lemmas but does not capture the NLTK release string. Candidates are embedded with the template “a photo of a {lemma}” and ranked by distance from the ID label bank alone, with no OOD data used at any point; the 10,000 most distant are retained. Original NegLabel instead includes nouns and adjectives, percentile-based mining, and grouping. The adaptation therefore cannot be interpreted as a faithful reproduction or a test of the full method. Its archive retains the SHA-256 digest of the selected bank and its first 50 entries, not the complete list, so exact independent regeneration depends on matching the corpus count above.

### 2.5. Statistical Scope and Evidence Levels

The primary results are descriptive AUROCs and empirical operating counts for frozen datasets/models. Where retained, bootstrap intervals resample ID and OOD examples within populations, using shared indices for detector contrasts (B=1000, seed 42). They characterize sample variability conditional on the fitted model and the assumed independent sampling units. They do not measure training-seed variability, route-to-route transfer, or unseen-domain risk. Driving-frame dependence and repeated spatial matches make frame-level intervals unsuitable as deployment guarantees; new real-world control tables therefore report point estimates and sample counts. Perfect sample separation is stated as an empirical value of 1.000 without converting a binomial rule of three into an AUROC confidence interval.

Additional controls were designed after reviewing the baseline, not preregistered. Multiple populations, prompts, readouts, and temperatures are reported together without a confirmatory family-wise significance claim. Subgroup and processing analyses are exploratory. For future generalization estimates, routes, drives, or acquisition units must be independently replicated; repeating inference on the same frames is a computational-repeatability test only. File S6 retains raw result archives for provenance but supplies separate descriptive tables: historical CI and in-sample-threshold fields are not all endorsed by this revision.

## 3. Score-Level Findings and Same-Encoder Controls

### 3.1. PUG Results and Noncausal Checkpoint Contrasts

Table 2 reports maximum-cosine performance for the three released checkpoints. CLIP ViT-B/32 has weak CS discrimination (0.504) while its SS AUROC is 0.660; the archived combined-shift score is 0.631. This is a property of the specified score, prompts, and sample. An interval containing 0.5 would not prove equivalence to random ranking, nor would an interval excluding zero for a checkpoint difference identify its cause.

The +0.021 CS contrast is between pretrained checkpoints, not a causally isolated loss effect. Matching a ViT-B/16 patch size leaves other training and architecture attributes uncontrolled. These measurements neither rule out a loss effect nor establish a universal architectural failure. A causal test would require interventions on one training factor with the data/recipe controlled and training replicated.

### 3.2. Text-Free Readouts of Frozen CLIP

Table 3 directly tests whether the same image encoder contains useful monitoring information. All four ID-fitted readouts are reported, including their weaker cells; no single readout is selected retrospectively as universally best. On PUG CS, preprojection Mahalanobis reaches 0.809 against 0.504 for maximum cosine. On BDD night, preprojection KNN reaches 0.913 against 0.534. Projected-space readouts also discriminate many of the shifts, so the evidence is not confined to features before text alignment.

These are empirical counterexamples to the claim that CLIP representations cannot register appearance changes. They establish accessible information for the tested readouts and reference samples, not the cause of that information or a universally optimal detector. ID-fitting requirements, feature-space scale and sampling remain relevant. The appropriate description of the baseline is *content-prompt score insensitivity on specified populations*; we withdraw the representation-wide “semantic anchoring blindness” interpretation.

**Figure 2:**
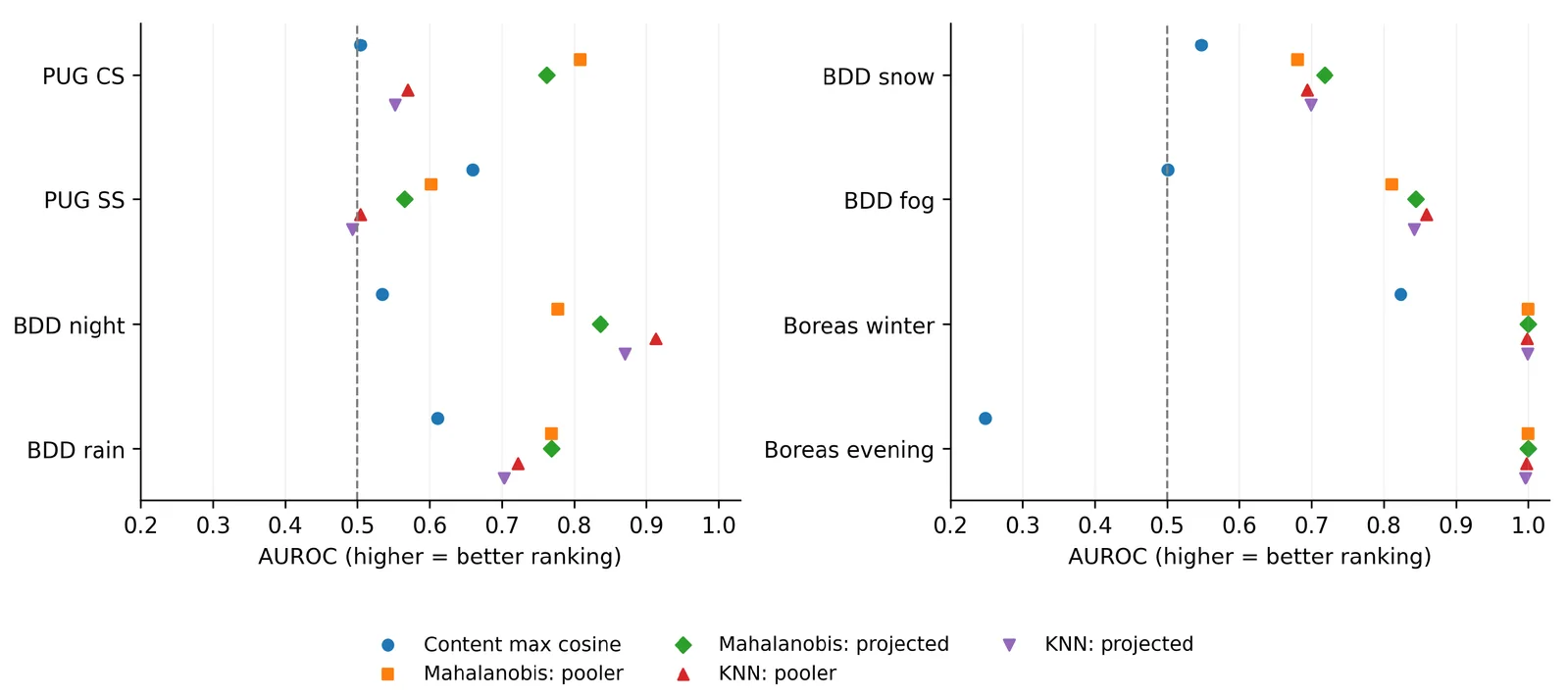
Readout dependence for the same CLIP encoder (Table 3). Maximum cosine and the four ID-fitted readouts use the same evaluation images. The dashed line indicates AUROC 0.5, not a statistical equivalence test.

Two of these rows establish less than they appear to. An unwhitened control—plain Euclidean distance to the ID statistics mean, fitted on the same split and using no information from the shifted population—already reaches 0.996 and 0.997 on Boreas winter and evening in the pooler space, against 1.000 for the fitted readout. Those two populations are therefore separated by a margin that almost any ID-fitted distance recovers, so they show that the shift is large rather than that the representation encodes it in a particular way. On PUG covariate shift, the same control gives 0.552 against 0.809, and on BDD rain, 0.636 against 0.769, so there, the fitted covariance carries the result. The counterexample to representation-wide insensitivity therefore rests on the PUG and BDD rows rather than on Boreas. One case reverses: on BDD night in the pooler space, the Euclidean control reaches 0.862 against 0.777 for the Mahalanobis readout, while the projected-space Mahalanobis value for the same population (0.836) is below it as well. We report this rather than select against it; it indicates that the unconditional whitening is not the best available use of this feature space on that population, which does not affect the comparison against the content-prompt baseline. The control values for all sixteen cells are in File S6. Figure 2 plots the content-prompt baseline against the four ID-fitted readouts for every population.

### 3.3. Prompt Content and Scoring Operation

Table 4 separates changes in prompt bank from the signed appearance contrast. Simply adding shifted-condition prompts to a *maximum* similarity score can increase similarity for those conditions: high similarity to “night” then counts as ID-like under the unchanged maximum-cosine direction. This explains why the operation does not by itself implement a clear-versus-shift alarm. The signed contrast encodes that decision explicitly and gives high discrimination for several named shifts, including 1.000 empirical AUROC on both Boreas populations. These results further contradict universal representation blindness, but they use shift-specific prior knowledge and are not a fair basis for claiming superior unknown-shift generalization.

The unrelated bank (violin, bookshelf, sandwich, telescope, and bassoon scene prompts) also varies substantially by population. It provides a negative-control diagnostic of incidental similarity structure, not a recommended monitor. The new prompt experiment covers BDD/Boreas; no analogous appearance-prompt sweep was completed on PUG, whose prompt-specific limitation remains explicit.

### 3.4. Alternative CLIP-Based Scores

MCM and the adapted negative-label score do not consistently recover the text-free results (Table 5). MCM at its default T=1 gives 0.569 on BDD night and 0.411 on Boreas evening. This describes the scores on the six scene prompts and monitored populations. It does not establish failure of MCM on its original semantic-OOD task or of the full NegLabel method. The same-encoder text-free and appearance-contrast controls already show that the broad claim about all VLM readouts would be false.

### 3.5. PUG Version Discrepancy and Inference Repeatability

The previous draft attributed an AUROC change from 0.504 to 0.5195 to Apple MPS floating-point nondeterminism. An image-path and score audit identifies a different explanation. The original RE09 archive has fixed-ID-high CS AUROC 0.5041465. The RE02 archive uses another ID sample and gives 0.480525 in the same direction; its analysis script chooses the larger of *A* and 1−A, producing 0.519475. The two archives share all 1000 CS image paths and exactly identical CS scores, but only 57 of 1000 ID image paths; all 57 shared ID scores are also identical. Thus, the cited discrepancy involves a changed ID sample and post hoc direction selection. We remove the nondeterminism attribution and the DeLong [32] p=0.131 value imported from that other analysis. File S8 provides both source archives and an independent fixed-direction calculation.

A further computational check repeats the full CLIP inference five times under the default settings and five times with deterministic execution requested, keeping the PUG draw fixed. Each mode reproduces CS 0.504146 and SS 0.659593 to six decimals, with bit-identical scores across repeats in the logged environment. This establishes observed repeatability for that configuration, not universal MPS determinism. The tiny last-digit difference from the older serialized archive does not affect the reported three-decimal values. Neither repeated inference nor a sample-bootstrap interval characterizes sampling uncertainty; that is measured separately below.

A completed resampling study now addresses the sampling question directly. The published sampling procedure was re-run with its fixed seed replaced by ten values (42–51), redrawing every population independently, and the full protocol was executed on each draw at n=1000 and again at n=5000 per population. Before any scoring, the seed-42 draw was verified to reproduce the published population membership exactly: the SHA-256 digest of the sorted file list matches for all five populations. The seed-42 cells then reproduce the archived values to within $1\times 10^{-6}$ AUROC across the eighteen published detector/population combinations, although each image is encoded once into a cache shared by all draws, so batch composition differs from the original run.

Three observations follow, and each is bounded by what ten draws can support. Table 6 reports the per-draw summary. First, the archived 0.504146 is an ordinary draw from this distribution: the ten-draw mean at n=1000 is 0.4962 with an observed range of [0.4799,0.5090], placing the archived value 0.77 sample standard deviations above the mean. The covariate-shift interval contains chance in all ten draws at both population sizes, so the chance-level reading does not depend on the particular draw. Second, for eleven of the eighteen detector/population combinations, the between-draw 1.96SD is smaller than the within-draw bootstrap half-width already reported, so those intervals are not narrower than the draw-to-draw dispersion; the unconditional Mahalanobis readout is the exception, exceeding it by factors of 1.21 on covariate shift and 1.74 on semantic shift, and Mahalanobis differences below roughly 0.02 AUROC should be read with that in mind. Third, increasing the population to n=5000 narrows the within-draw interval as resampling theory predicts (CLIP covariate half-width falls by 2.26× against the $\sqrt{5}=2.24$ expectation) but does not narrow the Mahalanobis between-draw dispersion proportionally (a factor of 1.2 against 3.8 for the logit-based scores). The Mahalanobis statistics are fitted on a fixed 500-image in-distribution split regardless of evaluation size, so that estimation component does not shrink with the evaluation population; at n=5000, its between-draw 1.96SD reaches 3.24 times the bootstrap half-width. Where a distance readout’s uncertainty matters operationally, enlarging the fitting split is the applicable lever, not the evaluation set. These draws still come from finite pools, so at n=5000, the draws overlap (the semantic-shift pool holds 7200 images in total) and the between-draw dispersion there is correspondingly compressed; no full-corpus claim is made.

Finally, the DeLong test [32] is restored for the archive the tables actually report, rather than left removed. On the archived seed-42 covariate-shift cell, it gives p=0.748 against AUROC =0.5, and its asymptotic interval [0.479,0.530] agrees with the reported stratified-bootstrap interval to three decimals. The previously printed p=0.131 belonged to the other archive discussed above and is not reinstated.

### 3.6. Natural Corruptions

CIFAR-10-C compares 2000 fixed CIFAR-10 test indices with their corrupted copies for 15 corruption types at severities 1, 3, and 5. CLIP uses the CIFAR class prompts and the same maximum-cosine scoring operation. The mean AUROCs in Table 7 show limited discrimination at lower severity and improvement for some severe corruptions. Per-corruption values for all 15 corruption types are given in Table A1 in Appendix A. The original ResNet18 Mahalanobis arm omitted the normalization used to train its standard checkpoint. The same-checkpoint preprocessing audit in File S9 reproduces the 45 original raw-input AUROCs within rounding tolerance and then refits/scores with the correct channel normalization. Its corrected severity means are 0.490, 0.527, and 0.624, replacing the original 0.438, 0.394, and 0.420. We therefore withdraw the explanation that persistent below-chance performance was a property of a small or fragile backbone. The images retain their source labels by construction, but no independent semantic-recognition or human-perceptibility control establishes that the variation across corruption types is caused by semantic destruction.

## 4. Driving-Population Monitoring and Acquisition Controls

### 4.1. Baseline BDD and Boreas Comparisons

**Figure 3:**
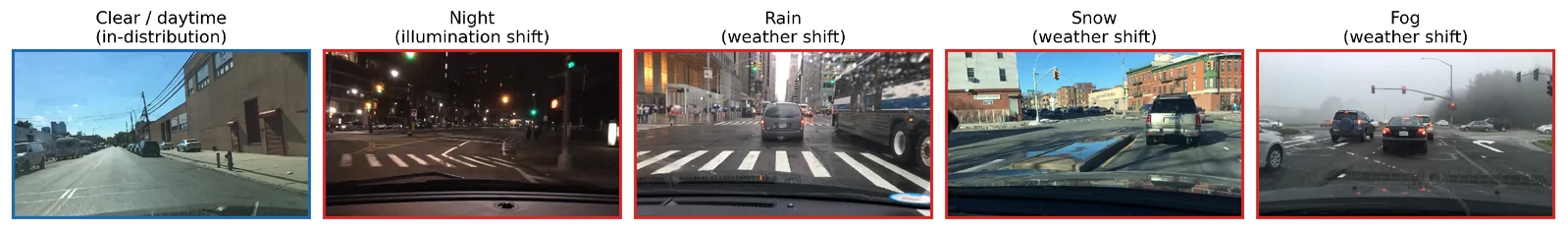
BDD100K monitoring populations. The reference distribution is clear daytime (blue); the four monitored departures are illumination and weather conditions labeled in the corpus metadata (red). All are ordinary road scenes containing the same object classes, so the monitored quantity is appearance rather than semantic novelty.

**Figure 4:**
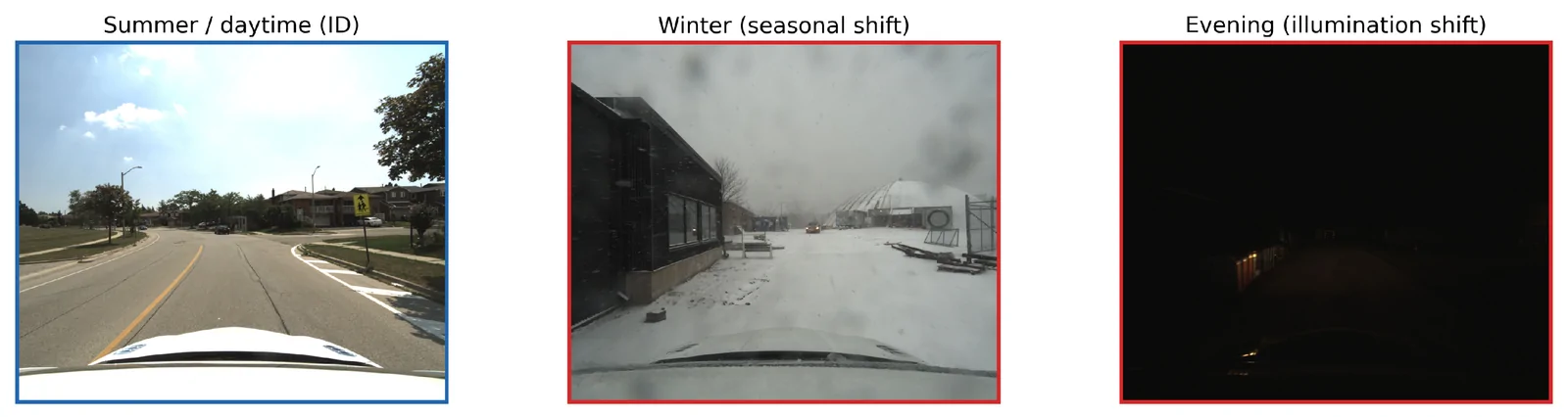
Boreas monitoring populations from repeated traversals of one route. Summer daytime frames are the reference (blue border); winter and evening are the monitored departures (red border). The content-prompt baseline ranks these two departures in opposite directions relative to the reference (Table 9).

Table 8 and Table 9 preserve the original driving-score baselines and frame lists. ResNet Mahalanobis has the largest AUROC among these five baselines on each population; the stronger CLIP readouts in Table 3 change the broader comparison. Maximum cosine is weak on BDD fog and has modest above-chance values on night and snow. In particular, BDD night 0.534 should not be described as statistically identical to 0.5: its archived conditional frame-bootstrap interval is approximately [0.506, 0.556]. Neither that interval nor its small point advantage establishes a useful operating point. Figure 3 and Figure 4 show example frames of the reference and monitored populations for the two corpora.

On Boreas, maximum cosine changes from AUROC 0.823 in winter to 0.249 in evening. With the ID-high convention fixed, evening frames tend to be ranked above the summer reference. This is *score sign instability across these populations*, not a general property of the CLIP representation. The corresponding operating behavior is measured next rather than inferred from AUROC.

### 4.2. A Fixed Threshold from Separate Summer Calibration

The 500-frame summer ECDF split in File S1 was reserved separately from the summer evaluation and shifted populations. Using its raw maximum-cosine scores, we set $\tau =s_{\left(26\right)}^{\mathrm{cal}}=0.24861189723014832$, the 26th ascending order statistic, which retains 475/500 calibration frames when equality is accepted. No winter/evening score enters threshold selection, and the direction is unchanged. Table 10 evaluates that one threshold on all populations.

This directly supports a narrow deployment-relevant statement: at this summer-fitted threshold, almost all sampled evening frames fail to trigger a distribution alarm, whereas just over half the winter frames trigger one. Acceptance is a missed monitoring event, not a certificate of safety or correctness. The separate calibration removes threshold reuse of the ID evaluation sample, but calibration and evaluation frames still come from one summer sequence. Temporal dependence, route coverage, and future conditions prevent treating the nominal 5% calibration target as a distribution-free or deployment-wide guarantee.

### 4.3. BDD Scene Composition and Night Heterogeneity

A scene-stratified diagnostic subsamples each BDD shifted population toward the scene proportions of the fixed ID evaluation set. Table 11 gives the resulting counts and AUROCs. Small remaining total-variation distances reflect finite stratum counts. This controls the measured scene mixture approximately; it does not match cameras, geography, traffic, exact location, or acquisition time. Persistence of a pattern after this control does not prove that weather alone caused it.

**Figure 5:**
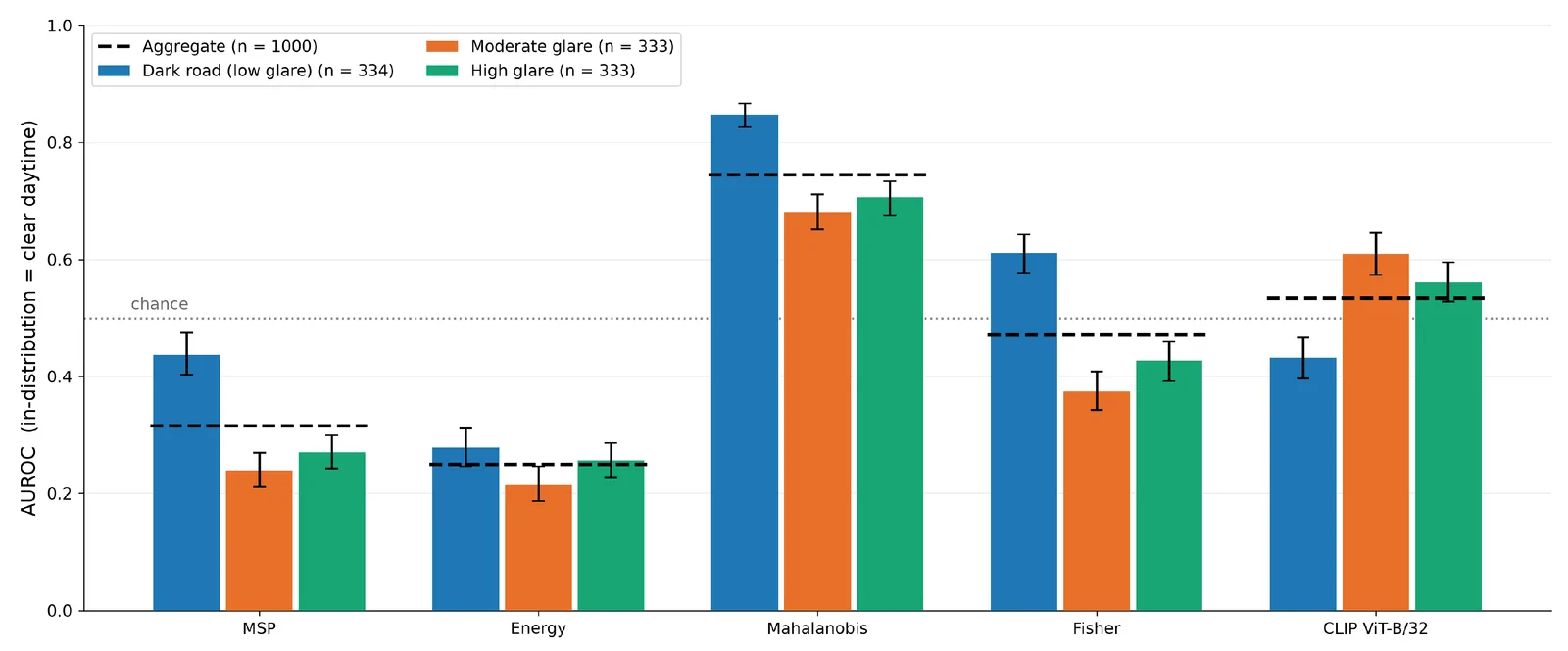
BDD night stratified by glare, with the aggregate value shown as a dashed line per detector. Bars are bootstrap 95% intervals. Aggregate night performance is not uniform within the population: the ResNet Mahalanobis readout separates dark-road frames best, whereas the maximum-cosine baseline is at its weakest exactly there.

The archived night subgroup analysis further describes within-population heterogeneity. A glare index combining saturated-pixel fraction, histogram bimodality, and concentration of the brightest 1% of pixels defines three tertiles: dark road (n=334), moderate glare (n=333), and high glare (n=333). Maximum-cosine AUROC is 0.432, 0.609, and 0.561, respectively; ResNet Mahalanobis gives 0.848, 0.680, and 0.706. The aggregate 0.534 and 0.745 values summarize this mixture and need not equal a subgroup value. These post hoc strata describe heterogeneity rather than causal glare effects or independent replications. Figure 5 shows the subgroup values against the aggregate for each detector.

### 4.4. Boreas Position and Heading Control

The original comparison did not spatially pair seasons. An additional winter control starts with the 500 summer evaluation frames and 14,129 winter candidates with global pose records. For each summer frame, the nearest winter position is selected first; the pair is then retained if its Euclidean separation and heading difference meet the tolerance. This is not a search over all heading-feasible candidates and not a one-to-one assignment. The strict 2 m/5-degree gate retains 243 summer frames but only 224 unique winter images (maximum reuse 12). The metadata audit behind this control, including the official test-split and reference-pose documentation, is in File S10. Median position and heading errors are 0.500 m and 0.482 degrees. Maximum-cosine AUROC is 0.761 and ResNet Mahalanobis AUROC is 0.996 on this weighted set of pairs.

A looser 5 m/10-degree diagnostic retains 487 summer frames and 345 unique winter images; one winter image is used 110 times. Its maximum-cosine/Mahalanobis AUROCs are 0.855/0.990. These estimates depend on the induced spatial sample and reuse weights; they are not 487 independent paired observations. We retain the complete pairing list and report these points without the original independent-frame bootstrap intervals. The magnitude change across tolerances shows why a shared route alone is insufficient to define a controlled comparison.

No equivalent evening spatial control was completed. The sequence boreas-2021-09-08-21-00 belongs to the official Boreas odometry and localization test splits; the official devkit documents that ground-truth poses are withheld for test sequences [33]. The local archive contains raw IMU and wheel-odometer records plus calibration, but no global reference camera poses. Thus, this is an expected public-reference-data limitation, rather than evidence of a failed download. Estimated trajectories derived from the sensor streams would constitute a separate localization experiment and would not provide an independent ground-truth spatial control. Accordingly, the evening sign result and fixed-threshold table remain statements about the time-sampled evening sequence. They do not isolate illumination from route segment, pose, or traffic composition. File S6 provides the pairing manifest and a distinct count of unique frames.

### 4.5. Camera–LiDAR Cross-Check

**Figure 6:**
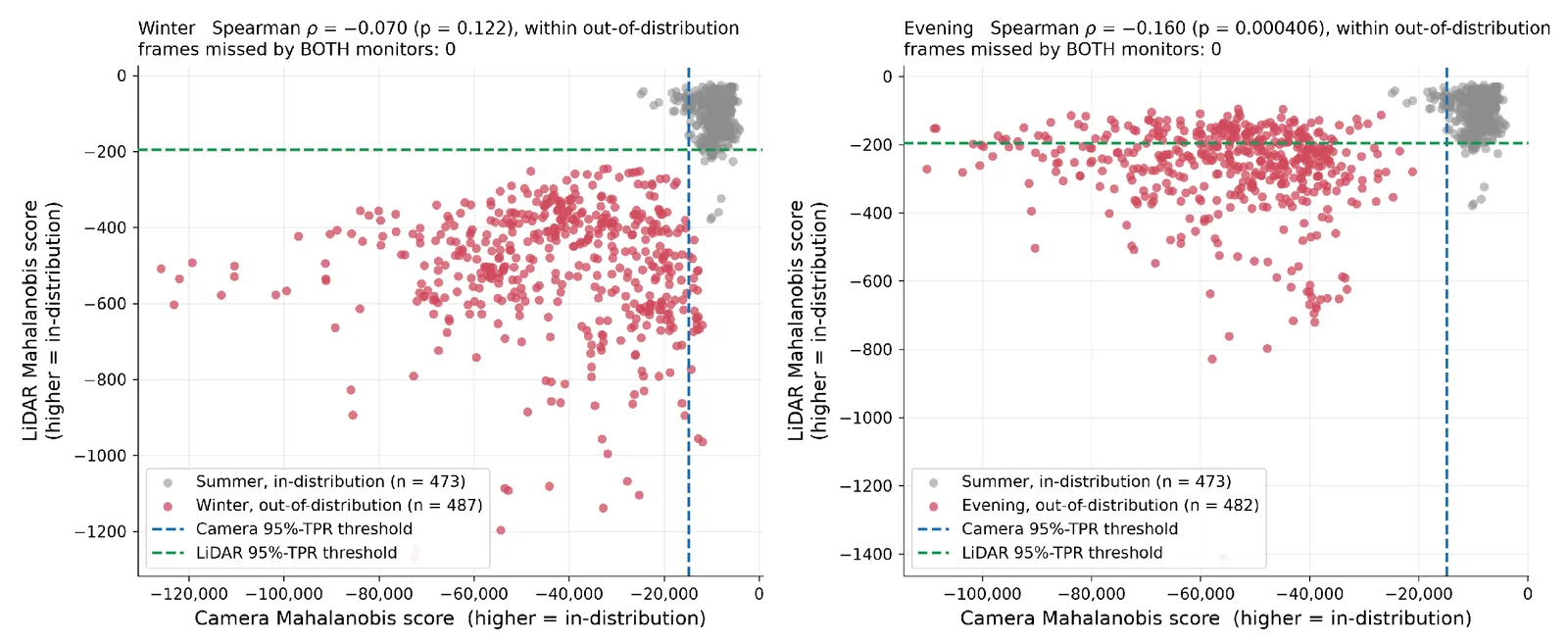
Camera and LiDAR Mahalanobis scores on the same Boreas frames, with each modality’s 95%-TPR threshold fitted on summer reference frames. No out-of-distribution frame falls in the retained quadrant of both monitors. The within-population rank correlation is weak, which is a necessary but not sufficient condition for error independence; no calibrated fused guarantee is claimed.

Camera frames and LiDAR sweeps in Boreas are matched by nearest timestamp with a 50 ms exclusion gate. This within-sequence cross-sensor synchronization is distinct from cross-season position matching. The retained evaluation counts are 473 summer, 487 winter, and 482 evening frames. The LiDAR baseline is an unconditional summer-ID-fitted Mahalanobis readout of 43 per-sweep features describing ring densities/intensities, height distribution, and intensity/range summaries, with covariance regularization $10^{-3}I$. Table 12 reports both modalities at a common operating point, and Figure 6 shows the paired per-frame scores.

Within the shifted populations, camera/LiDAR Mahalanobis score rank correlations are −0.070 in winter and −0.160 in evening. They are weakly negative observations, not evidence of statistical independence. At the two evaluation-fitted thresholds, no sampled shifted frame is missed by both Mahalanobis monitors. This finite-sample complementarity motivates a separately calibrated fusion experiment but does not guarantee a fused miss rate, establish independent errors, or certify safety. These detectors measure distribution departure; physical sensor degradation and downstream task error are different quantities.

### 4.6. Sensitivity to the Imaging Chain

**Figure 7:**
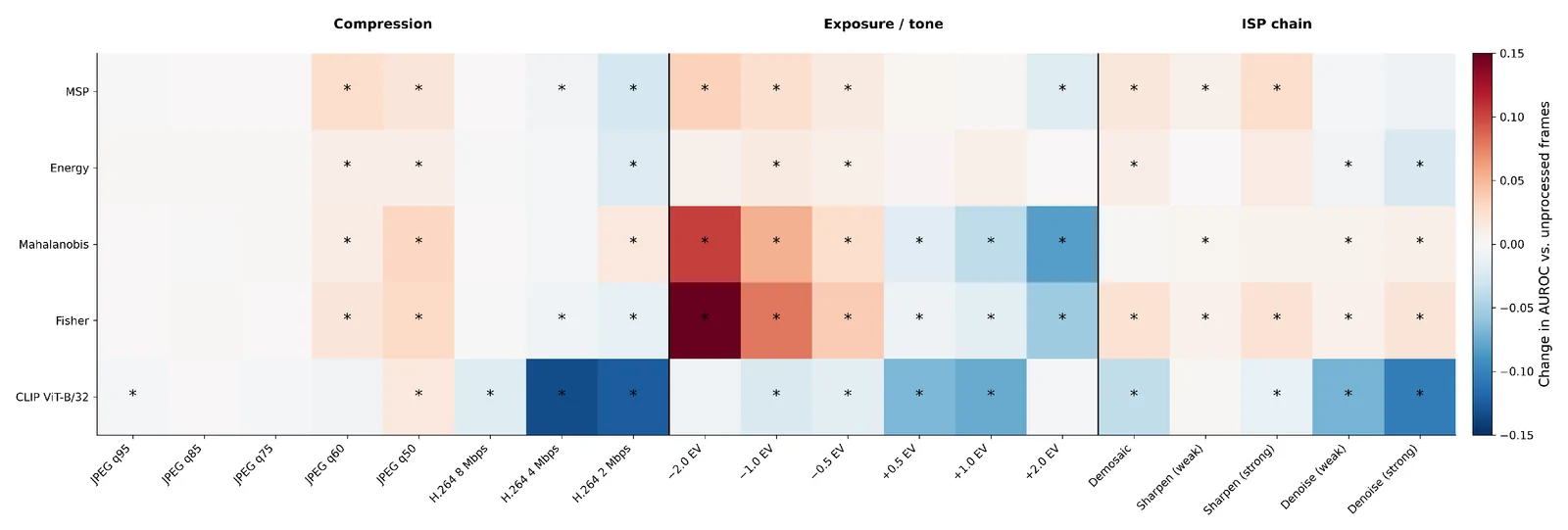
Change in detection AUROC relative to unprocessed frames across nineteen imaging-chain variants (Table 13). Asterisks mark intervals excluding zero without multiplicity correction; the comparisons are exploratory. Exposure shifts move the ID-fitted readouts most, while the maximum-cosine baseline is most sensitive to aggressive video compression.

The archived postprocessing study applies JPEG quality 95, 85, 75, 60, and 50; H.264 at 8, 4, and 2 Mbps; tone shifts of ±0.5, ±1, and ±2 EV in linear light; and re-mosaicing/bilinear reconstruction, two sharpening strengths, and two bilateral-denoising strengths. Both ID and shifted evaluation populations receive each variant; Gaussian statistics and Fisher ECDFs remain fitted on unprocessed ID frames. Table 13 reports the maximum absolute AUROC movement across the six driving populations and levels of each axis. The per-variant values and the archived per-sample score arrays are in File S3. Figure 7 resolves these movements by individual variant for the BDD night population.

Most nominally significant CLIP changes on BDD were negative (46 of 48 archived paired-bootstrap exclusions of zero). These are exploratory, unadjusted comparisons across many levels. They show processing sensitivity, not a representation mechanism or codec invariance of a comparator. For example, an E8 retrospective fixed-baseline-threshold calculation gives a Boreas winter maximum-cosine miss rate increase from 0.462 to 0.756 at JPEG quality 50. Its threshold was fitted on unprocessed ID *evaluation* scores; it is an in-sample reference sensitivity analysis and must not be confused with Table 10. On BDD night, the baseline miss rate was already 0.945, so substantial AUROC changes can coexist with only small further miss-rate increases.

Neither dataset supplies camera RAW in this study. BDD source JPEGs and Boreas 8-bit post-ISP PNGs have already passed through acquisition processing. Re-mosaicing is a second synthetic pass, and tone mapping cannot reproduce exposure-dependent photon noise or recover clipped highlights. Thus, the experiment does not isolate physical exposure or the native sensor ISP. Both the processing settings and threshold require validation for the intended monitor; the measurements do not justify leaving codec settings unconstrained.

## 5. Preprocessing Audit and Training Sensitivity

This section reports a correction rather than a new capability. Section 5.1 documents why the earlier adversarial-training result was withdrawn because the corrected numbers replace values that were previously circulated and the reason for the change has to be auditable; Section 5.2 is the replicated training study that takes its place. A reader interested only in the monitoring findings can proceed to Section 6.2.

### 5.1. Correction of the Original Checkpoint Comparison

The original standard/PGD-3/PGD-10 comparison used ToTensor() inputs for all three saved ResNet18 checkpoints. Source inspection shows that the standard checkpoint’s training path instead normalizes channels with mean (0.4914, 0.4822, 0.4465) and standard deviation (0.2470, 0.2435, 0.2616). The PGD-3 and PGD-10 paths use unnormalized [0, 1] tensor inputs. Applying an identical preprocessing function to these differently trained checkpoints was therefore not a fair comparison.

An A/B audit reconstructs the original detector suite and reproduces all 24 archived AUROC cells under raw inputs. It then refits the ID statistics and evaluates each checkpoint under its training-matched preprocessing. The standard checkpoint’s full CIFAR-10 test accuracy changes from 41.55% to 90.24%; the raw-input PGD-3 and PGD-10 accuracies are 77.35% and 62.49%. Applying the standard normalization to the adversarial checkpoints would also be a mismatch (46.16% and 42.77% accuracy). Thus, the correction is checkpoint-specific, not a blanket input transformation.

Table 14 replaces the original AUROC table. The same CIFAR-specific ResNet18 stem, class-conditional Mahalanobis statistics (500 examples per class, tied covariance plus $10^{-3}I$), KNN bank of 5000 ID training examples and full test populations are used: CIFAR-10 10,000, SVHN 26,032, and CIFAR-100 10,000 [34,35]. Raw-image data and checkpoints were locally available for the audit; File S9 records the scripts, results, and checkpoint hashes. An independent full-data CPU rerun of the normalized standard checkpoint reproduces its eight corrected AUROCs within $5.1\times 10^{-7}$ of the archived values and its 90.24% accuracy; its per-sample scores are included in File S9. Corrected intervals for all checkpoint arms were not recomputed, so we report point estimates without reusing the invalid comparison’s intervals.

For near-OOD Energy, the standard AUROC is now 0.869 rather than 0.543; PGD-3 remains 0.759. The alleged +21.6-point gain becomes a descriptive −11.0-point contrast against the correctly evaluated standard checkpoint. All eight standard-versus-PGD-3 detector/population contrasts favor standard in this corrected archive. We withdraw the original AT–OOD synergy and inverted-U claims. This does not prove that adversarial training universally reduces OOD performance: the checkpoints differ in training history, and the linked standard-model source specifies 15 epochs whereas the adversarial scripts specify ten. The original “matched ten-epoch budget” description was unsupported for these saved models; weights alone do not recover their full run history.

### 5.2. Separate Five-Seed Training Study

A completed follow-up trains five seeds (42–46) for each of the 0, 3, 5, 7, and 10 training-PGD steps, at a ten-epoch budget. It uses SGD (initial learning rate 0.1, momentum 0.9, weight decay $5\times 10^{-4}$), a completed cosine schedule, batches of 128, random crops/flips, and raw tensor inputs for every configuration. PGD [36], the iterated form of the single-step sign attack [37], uses $L_{\infty }$ radius 8/255, step size 2/255, random initialization, and the stated number of steps. A 48,000-image CIFAR-10 subset fits network weights; 2000 training images are reserved from those updates. The OOD scores use the full test sets and a 5000-image training reference draw per seed.

This follow-up is a separate implementation, not a literal extension of the old checkpoints: it uses torchvision’s default ResNet18 stem (7-by-7 stride-2 convolution plus max pooling) rather than the original CIFAR-specific 3-by-3 stride-1 stem with no max pool, and an unconditional Gaussian rather than the archived class-conditional readout. Its failed gate against the original numbers is retained in File S9. The seed-level results in Table 15 characterize this explicit new protocol; they must not be spliced into the corrected checkpoint table as one experiment.

**Figure 8:**
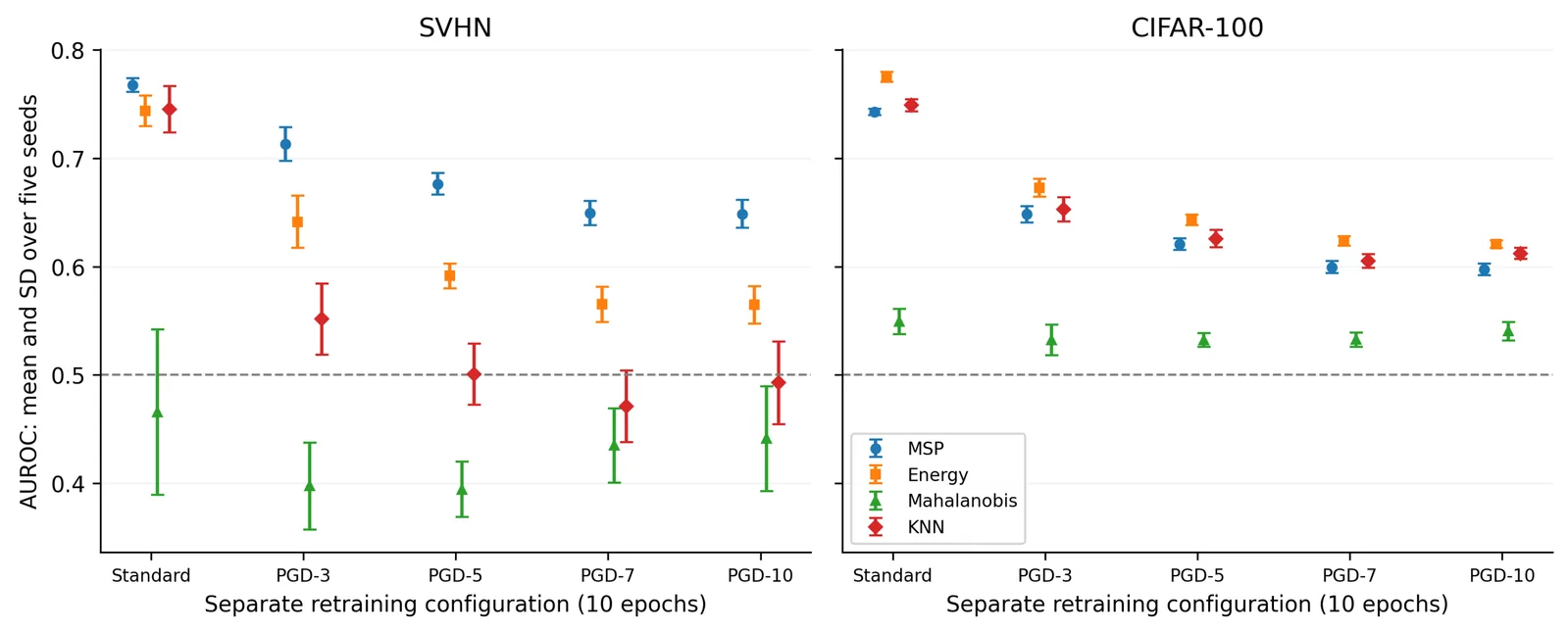
Separate retraining protocol: categorical configuration means with standard-deviation bars across five training seeds. All use ten epochs; the categories do not define a controlled continuous strength axis.

For example, CIFAR-100 Energy averages 0.775 for standard and 0.673 for PGD-3. Figure 8 shows the same configuration means with their between-seed standard deviations. This follow-up also fails to support the original benefit, but equal epochs still do not equalize compute, optimization difficulty, or attained accuracy. The direction of the association is the one the robustness literature would predict: adversarial training reshapes the learned features away from the non-robust components that carry much of the standard predictive signal [38], and the accompanying clean-accuracy cost is intrinsic to the objective rather than an artifact of this budget [39]. The scope of the present finding is one training objective; the robustness–accuracy trade-off can be traded differently [40], and training-time interventions aimed at the detection task itself, such as outlier exposure, are a separate family whose effect on OOD detection is not tested here [41]. We do not infer an optimal attack strength or a universal response curve from these configurations.

A further seed-42 budget sweep trains PGD-3 and PGD-10 for 10, 20, and 30 epochs, completing a cosine schedule separately for each budget. Table 16 reports these exploratory points. Clean accuracy is measured on the full 10,000-image CIFAR-10 test set and the PGD-20 single-restart robust accuracy on 2000 held-out training indices passed through the test transform, so neither column carries augmentation noise; they still describe different populations, and the sweep remains an exploratory single-seed probe rather than a matched-accuracy control. No stopping rule or hyperparameter choice is claimed to have been validated on an independent final test.

Both accuracy columns have since been remeasured on proper populations. Clean accuracy is now the full 10,000-image CIFAR-10 test set rather than the first eight batches, and PGD-20 robust accuracy uses the same 2000 held-out indices through the test transform, so no training augmentation enters the measurement. The remeasurement moves individual clean-accuracy values by at most 0.018 (mean −0.005) and does not change any ordering. Table 16 reports the remeasured values; the seed-averaged accuracies for the five-seed protocol are standard 0.776 ± 0.006, PGD-3 0.597 ± 0.014, PGD-5 0.542 ± 0.010, PGD-7 0.504 ± 0.011, and PGD-10 0.503 ± 0.011 clean, against 0.001, 0.285, 0.316, 0.316, and 0.322 robust.

With comparable accuracy measurements available for every model, the two studies can be summarized jointly as a descriptive association. Pooling the 25 five-seed models and the 6 budget-sweep models gives 31 models spanning clean accuracies 0.49–0.78, training-PGD steps 0–10, and budgets of 10–30 epochs. Regressing AUROC on clean accuracy and then adding the step count (Table 17) shows that, for the confidence-, logit- and neighbour-based scores, clean accuracy accounts for most of the between-model variation, and the step count adds little beyond it: its fitted effect across the entire 0-to-10 range is at most 0.011 AUROC in absolute value. That residual is statistically detectable for two of the eight combinations under an *F* test (F=22.0 for MSP and F=7.3 for Energy on near-OOD, against $F_{1,28}^{0.05}=4.20$), so it is not zero; it is small relative to the accuracy term, whose fitted slope is roughly 0.5–0.9 AUROC per unit of clean accuracy over a 0.29-wide accuracy range.

The unconditional Mahalanobis readout does not follow this pattern at all: clean accuracy accounts for essentially none of its variation ($R^{2}=0.073$ near-OOD and 0.004 far-OOD), and neither does the step count. Within this pool, it behaves as a measurement that does not track classification quality, which is consistent with the driving-population results of Section 4, where it is the readout that retains discrimination when the text-aligned scores do not. We report this as an observed dissociation, not as an explanation of it.

These regressions do not license a causal reading. Training-attack strength, optimization budget, and attained accuracy move together by construction here, and a third factor could drive both clean accuracy and OOD ranking; the pool also covers one architecture, one in-distribution dataset, and one family of accuracy-reducing interventions. What the summary supports is narrower and negative: within these 31 models, the training-PGD step count carries little information about detection AUROC once clean accuracy is included, so reporting an attack-strength response curve without the corresponding accuracies can present an accuracy difference as a training effect.

The budget results reinforce the need to separate training configuration, fitting quality, and OOD ranking. They do not establish that clean accuracy causes AUROC or that conditioning on clean accuracy identifies a causal training effect. The robustness–accuracy question [39] and the distribution-monitoring question remain distinct.

## 6. Discussion and Practical Limits

### 6.1. Implications for a Monitoring Pipeline

The readout controls change the interpretation of the original negative result. Low maximum-cosine AUROC identifies a weak combination of prompt banks and scores for the defined monitoring task; it does not show that CLIP cannot encode the relevant condition. ID-fitted geometry and shift-informed text contrasts can extract useful information from the same encoder. Their additional reference data or named-condition knowledge must be counted when comparing them with zero-shot content scoring.

A practical evaluation should specify the target event, freeze score direction, fit a threshold on the intended reference distribution, and measure both false alarms and misses across relevant conditions. The Boreas operating counts illustrate why a high AUROC on one shift cannot validate a monitor on another. Fusing scores also needs direct validation: the uniform empirical Fisher baseline performs below the strongest original constituent on the studied driving populations, and no independence or best-constituent guarantee follows from its formula. A conformal treatment of the same fusion problem, which supplies the coverage guarantee the uniform baseline lacks, is developed separately [42]; the present result should be read as a caution about the unweighted formula rather than about score fusion in general.

The requirements above are the ones a fielded monitor has to satisfy, and they are stricter than the benchmark protocol the original analysis followed. Deployment-side monitors of this kind have been built for 2D object detection under a real-time latency budget [43], and the same encoder–score–prompt–calibration separation applies wherever a learned perception model is monitored on a different imaging modality, such as robustness-oriented X-ray inspection workflows [44]; those settings are cited here to place the requirement, not as evidence for the present measurements. The adversarial-robustness face of the same evaluation problem—whether a standard classification-targeted attack measures what an adaptive detector-targeted attack would—is examined in a companion study on the same benchmark suite [45], which is under review in parallel and shares the PUG and BDD100K frame lists but no result reported here.

### 6.2. Embedded Measurements

The original NVIDIA Jetson AGX Orin (NVIDIA Corporation, Santa Clara, CA, USA) measurements are retained in Table 18. Batched timings are elapsed processing time divided by batch size; they are not per-frame end-to-end response latencies including buffering, sensor acquisition, decoding, or transport. A separate batch-8 run of the ResNet50-plus-Mahalanobis monitor records approximately 331 frames/s and 11.0 W over 600 s, with no reported clock throttling in that log. The timings, platform record and thermal trace are in File S5. These are measurements of the tested workload and platform, not a power or throughput guarantee for a deployed vehicle. The platform record further discloses that the timing Gaussian parameters were fitted on 100 bundled BDD frames to reproduce the canonical feature/class dimensions; two unavailable scene means were filled with the global mean. Those shape-matched timing parameters were not the 3000-frame detection-fit parameters and must not be used for detection accuracy. Thus, this benchmark establishes processing cost at the recorded shapes, not end-to-end validation of the exact accuracy pipeline.

The full-monitor batch-1 value of 13.45 ms is about 4.51 times the batch-8 per-image cost of 2.98 ms. Batch size therefore affects processing efficiency, but batching is not logically mandatory for real-time use at every target rate. The additional CLIP text-free, prompt-contrast and negative-label readouts introduced in this revision were not benchmarked on the Jetson. Their memory and latency cannot be inferred from the original ResNet monitor run.

### 6.3. Remaining Limitations

The model scope remains CLIP ViT-B/32, two additional PUG checkpoints, and small ResNet baselines. The same-encoder controls refute a universal incapacity claim within this scope but do not characterize every larger or differently trained VLM. Hyperparameters and prompt variants are exploratory; the negative-label adaptation has an incomplete selected-label archive. The primary PUG results use one 1000-image draw per evaluation population. The ten-draw resampling study of Section 3.5 is a sensitivity analysis layered on that primary draw, at n=1000 and n=5000 per population; it bounds draw-to-draw dispersion but does not replace the primary draw, and every PUG value reported outside Table 6 comes from it. PUG appearance-prompt sensitivity and full-corpus variability remain unmeasured.

BDD scene stratification does not control camera or geography. Boreas contains temporally dependent frame samples from few sequences, and nearest-position matching reuses some winter images heavily. The evening test sequence lacks publicly released reference poses and therefore an equivalent ground-truth spatial control. The fixed-threshold experiment is frame-disjoint but not an independent multi-drive calibration study. Strong monitoring generalization requires new acquisition units and frozen protocols. Both corpora capture structured on-road driving; off-road autonomy relies on a distinct and more heterogeneous set of benchmarks [46], over which a much broader reference manifold would change the behavior of every detector studied here. No downstream perception loss, physical safety event, or clinical outcome is measured here.

One quantity in Table 3 is regularization-dependent and is reported together with its convention for that reason. The projected 512-dimensional embeddings are unit-norm, so the fitted covariance is poorly conditioned (condition number $\approx 1.1\times 10^{9}$ on the BDD statistics split) and the absolute $+10^{-3}I$ shrinkage used throughout therefore acts as a strong regularizer relative to the data scale. Substituting a trace-relative shrinkage of the same nominal size moves the projected Mahalanobis AUROC by up to 0.111 (BDD night: 0.836 against 0.725), whereas the pooler-space values move by at most 0.0014 under the same substitution. The qualitative conclusion is unaffected—every text-free readout remains far above the maximum-cosine baseline under either convention—but the projected-space figures should be compared only against the stated convention.

Sensor-chain ablations operate on already processed images and involve many exploratory comparisons. The camera–LiDAR zero joint miss observation does not establish independent errors or a calibrated fused guarantee. The corrected original training comparison still uses one saved checkpoint per configuration with nonmatched history. The separate five-seed retraining provides between-seed variation, but uses a different stem/readout and does not match convergence or compute. The one-seed budget sweep and augmented validation accuracy do not eliminate this limitation. The present paper contains no standard-versus-adaptive attack evaluation-gap result.

## 7. Conclusions

On the evaluated camera populations, monitoring behavior depends strongly on how a frozen vision–language representation is scored. The original maximum-cosine weakness is not evidence of representation-wide blindness: same-encoder Mahalanobis/KNN and shift-informed prompt contrasts recover substantial discrimination. Released CLIP/SigLIP checkpoint differences are descriptive and do not isolate the training loss. A separate audit finds a preprocessing mismatch behind the original adversarial-training advantage; corrected checkpoint evaluation and a five-seed follow-up do not support that advantage.

For the content-prompt baseline, the Boreas summer-calibrated threshold misses 44.8% of sampled winter and 99.6% of evening frames, with 5.8% false alarms on held-out summer frames. This measured operating-point limitation remains meaningful under the stated observational sampling design. Scene and position controls qualify the interpretation without removing all acquisition confounding. Future studies should evaluate complete encoder–score–prompt–calibration pipelines with independent drives, explicit target events, and replicated training where training effects are claimed. 

## Figures and Tables

**Table 1:** Evaluation and ID fitting counts. Each reported driving comparison uses the same ID evaluation population within its dataset.

Dataset/Role	ID Statistics	ID Calibration	ID Evaluation	Monitored Populations
PUG controls	500	—	1000	CS 1000; SS 1000
BDD100K	3000	1000	1000	Night 1000; rain 396; snow 422; fog 143
Boreas	500	500	500	Winter 500; evening 500

**Table 2:** PUG maximum-cosine checkpoint comparison, identical 1000-image evaluation populations. Contrast intervals are paired sample-bootstrap 95% intervals, conditional on the fixed checkpoints. Shared patch size does not control the pretraining corpus, text encoder, optimizer, or preprocessing.

Model/Contrast	CS	SS
CLIP ViT-B/32	0.504	0.660
CLIP ViT-B/16	0.493	0.637
SigLIP ViT-B/16	0.514	0.637
SigLIP_16_ minus CLIP_16_	+0.021[0.005,0.036]	+0.000[−0.016,0.017]
CLIP_16_ minus CLIP_32_	−0.011[−0.029,0.008]	−0.022[−0.040,−0.005]

**Table 3:** Same frozen CLIP ViT-B/32 encoder, fixed ID-high score direction. M: unconditional Mahalanobis; K: 50th-neighbor cosine distance; pre: 768-dimensional pooler; proj: 512-dimensional L2-normalized projection. ID statistics and evaluation images are disjoint. Counts are in Table 1; text-free methods additionally fit an ID reference. The two PUG rows are independent generator draws, so bootstrap 95% intervals under the Section 2.5 convention (B=1000, seed 42, resampling indices shared across detectors) are given for them: PUG CS 0.809[0.789,0.828], 0.762[0.740,0.783], 0.570[0.546,0.594], 0.552[0.526,0.576]; PUG SS 0.602[0.576,0.624], 0.566[0.540,0.591], 0.504[0.478,0.529], 0.493[0.466,0.520], in the M (pre), M (proj), K (pre), K (proj) column order. The driving rows follow the point-estimate policy of Section 2.5 because their frames are not independent.

Population	Max Cosine	M (Pre)	M (Proj)	K (Pre)	K (Proj)
PUG CS	0.504	0.809	0.762	0.570	0.552
PUG SS	0.660	0.602	0.566	0.504	0.493
BDD night	0.534	0.777	0.836	0.913	0.870
BDD rain	0.611	0.769	0.769	0.722	0.703
BDD snow	0.547	0.680	0.718	0.694	0.699
BDD fog	0.501	0.811	0.845	0.859	0.843
Boreas winter	0.823	1.000	1.000	0.999	0.999
Boreas evening	0.249	1.000	1.000	0.998	0.996

**Table 4:** Prompt and scoring diagnostics on cached projected CLIP embeddings. The first four columns use maximum cosine; the final column uses Equation (4). Counts and wording differ across banks, and the appearance alternatives name the test conditions.

Population	Content (6)	Appearance (5)	Composed (30)	Unrelated (5)	Contrast (5)
BDD night	0.534	0.359	0.312	0.798	0.989
BDD rain	0.611	0.282	0.403	0.517	0.964
BDD snow	0.547	0.487	0.444	0.519	0.840
BDD fog	0.501	0.244	0.304	0.688	0.985
Boreas winter	0.823	0.202	0.214	0.881	1.000
Boreas evening	0.249	0.001	0.061	0.335	1.000

**Table 5:** Alternative scores on the same six scene ID prompts. All tested temperatures are shown. The final column is a NegLabel-inspired ungrouped noun-only adaptation, with implementation differences specified in Section 2.4.

Population	Max Cosine	MCM T=0.01	MCM T=0.1	MCM T=1	Negative-Label Adaptation
BDD night	0.534	0.536	0.564	0.569	0.348
BDD rain	0.611	0.531	0.548	0.552	0.471
BDD snow	0.547	0.504	0.481	0.477	0.394
BDD fog	0.501	0.491	0.516	0.520	0.417
Boreas winter	0.823	0.624	0.616	0.611	0.201
Boreas evening	0.249	0.412	0.410	0.411	0.348

**Table 6:** Ten-draw PUG resampling, seeds 42–51. “Between-draw SD” is the sample standard deviation of the AUROC across the ten independent draws; “bootstrap half-width” is the mean half-width of the within-draw stratified bootstrap interval that the tables otherwise report. The last column counts draws whose within-draw interval contains 0.5. These are descriptive dispersion measures, not confidence statements about the full corpus.

Model	Detector	Shift	*n*	Ten-Draw Mean	Between-Draw SD	Bootstrap Half-Width	CI Covers 0.5
CLIP ViT-B/32	max-cosine	CS	1000	0.4962	0.0103	0.0253	10/10
CLIP ViT-B/32	max-cosine	SS	1000	0.6533	0.0153	0.0240	0/10
ResNet50	Energy	CS	1000	0.7924	0.0083	0.0195	0/10
ResNet50	Mahalanobis	CS	1000	0.8853	0.0087	0.0141	0/10
ResNet50	Energy	SS	1000	0.9526	0.0040	0.0082	0/10
ResNet50	Mahalanobis	SS	1000	0.9649	0.0066	0.0074	0/10
CLIP ViT-B/32	max-cosine	CS	5000	0.4969	0.0021	0.0112	10/10
CLIP ViT-B/32	max-cosine	SS	5000	0.6540	0.0026	0.0106	0/10
ResNet50	Energy	CS	5000	0.7915	0.0031	0.0087	0/10
ResNet50	Mahalanobis	CS	5000	0.8848	0.0043	0.0064	0/10
ResNet50	Energy	SS	5000	0.9519	0.0010	0.0038	0/10
ResNet50	Mahalanobis	SS	5000	0.9638	0.0055	0.0033	0/10

**Table 7:** CIFAR-10-C mean AUROC across 15 corruption types; 2000 matched image pairs. CLIP is unchanged. The ResNet18 column now uses the training-matched channel normalization. The raw-input historical column is shown only to expose the correction.

Severity	CLIP Max Cosine	ResNet M: Corrected	ResNet M: Old Mismatch
1	0.494	0.490	0.438
3	0.523	0.527	0.394
5	0.610	0.624	0.420

**Table 8:** BDD100K observational weather/time strata, ID clear daytime (n=1000). ResNet50 weights are frozen; Mahalanobis is scene-conditional, and Fisher combines MSP, Energy, and this distance score. CLIP means content-prompt maximum cosine. Attribute filtering does not remove scene, camera, or geographic confounding.

OOD Population	MSP	Energy	Mahalanobis	Fisher	CLIP
Night (clear), n=1000	0.315	0.250	0.745	0.471	0.534
Rain (daytime), n=396	0.439	0.510	0.665	0.597	0.611
Snow (daytime), n=422	0.458	0.493	0.618	0.552	0.547
Fog (all), n=143	0.392	0.355	0.734	0.551	0.501

**Table 9:** Boreas original time-sampled populations, 500 summer ID, and 500 shifted frames per comparison. A repeated platform/route reduces some acquisition differences but these original samples are not position-paired. Mahalanobis is the unconditional ResNet50 readout.

OOD Population	MSP	Energy	Mahalanobis	Fisher	CLIP
Winter	0.122	0.204	0.994	0.925	0.823
Evening	0.516	0.191	1.000	0.980	0.249

**Table 10:** Fixed raw CLIP maximum-cosine threshold τ=0.24861189723014832, alarm s<τ, equality accepted. All populations have 500 frames. Calibration rates are in-sample; the remaining rates use disjoint frame IDs.

Population/Role	Alarms	Retained	ID False Alarm	Shift Miss Rate
Summer calibration	25/500	475/500	5.0% (fit)	—
Summer evaluation	29/500	471/500	5.8%	—
Winter evaluation	276/500	224/500	—	44.8%
Evening evaluation	2/500	498/500	—	99.6%

**Table 11:** Scene-stratified BDD sensitivity analysis; 1000 unchanged ID evaluation images. TVD is total-variation distance between the selected OOD scene mixture and ID scene mixture.

Population	Selected Shift *n*	Scene TVD	CLIP Max Cosine	ResNet Mahalanobis
night	455	0.0005	0.513	0.757
rain	285	0.0015	0.601	0.669
snow	228	0.0021	0.527	0.646
fog	125	0.0040	0.512	0.761

**Table 12:** Camera–LiDAR comparison on synchronized frames. Benchmark FPR95 uses empirical 95% ID retention on the restricted ID evaluation scores, unlike the separate-calibration experiment in Table 10.

Population	Sensor/Score	ID/Shift *n*	AUROC	FPR95
Winter	Camera ResNet Mahalanobis	473/487	0.994	0.041
Winter	Camera CLIP max cosine	473/487	0.827	0.466
Winter	LiDAR Mahalanobis	473/487	0.999	0.000
Evening	Camera ResNet Mahalanobis	473/482	1.000	0.000
Evening	Camera CLIP max cosine	473/482	0.251	0.996
Evening	LiDAR Mahalanobis	473/482	0.924	0.309

**Table 13:** Observed maximum absolute AUROC change over population-by-level combinations. ResNet Mahalanobis is unconditional in this ablation on both datasets. A smaller observed change does not establish invariance.

Axis	Population–Level Combinations	CLIP Max Cosine	ResNet Mahalanobis
JPEG/H.264	48	0.143	0.031
Exposure-like tone mapping	36	0.226	0.103
Second ISP-like pass	30	0.104	0.015

**Table 14:** Corrected original checkpoint comparison. Standard uses its training normalization; PGD-3 and PGD-10 use their training-matched raw tensor inputs. This repairs preprocessing but does not create a controlled equal-budget training intervention.

OOD Set	Detector	Standard: Normalized	PGD-3: Raw	PGD-10: Raw
SVHN	MSP	0.870	0.832	0.751
SVHN	Energy	0.857	0.820	0.684
SVHN	Mahalanobis	0.894	0.305	0.450
SVHN	KNN	0.915	0.777	0.622
CIFAR-100	MSP	0.845	0.739	0.659
CIFAR-100	Energy	0.869	0.759	0.676
CIFAR-100	Mahalanobis	0.619	0.542	0.535
CIFAR-100	KNN	0.862	0.745	0.667

**Table 15:** Separate ten-epoch retraining protocol: AUROC mean ± sample standard deviation over five seeds. These are between-seed standard deviations, not confidence intervals. Architecture and Gaussian differences from the old checkpoint study are disclosed in the text.

OOD Set	Detector	Standard	PGD-3	PGD-5	PGD-7	PGD-10
SVHN	MSP	0.768 ± 0.006	0.713 ± 0.016	0.676 ± 0.010	0.649 ± 0.011	0.649 ± 0.013
SVHN	Energy	0.744 ± 0.014	0.641 ± 0.024	0.591 ± 0.011	0.565 ± 0.016	0.565 ± 0.017
SVHN	Mahalanobis	0.466 ± 0.076	0.398 ± 0.040	0.394 ± 0.026	0.435 ± 0.034	0.441 ± 0.049
SVHN	KNN	0.745 ± 0.022	0.552 ± 0.033	0.501 ± 0.028	0.471 ± 0.033	0.493 ± 0.038
CIFAR-100	MSP	0.743 ± 0.003	0.648 ± 0.008	0.621 ± 0.005	0.600 ± 0.006	0.598 ± 0.005
CIFAR-100	Energy	0.775 ± 0.005	0.673 ± 0.008	0.643 ± 0.005	0.624 ± 0.004	0.621 ± 0.003
CIFAR-100	Mahalanobis	0.549 ± 0.012	0.532 ± 0.014	0.532 ± 0.006	0.533 ± 0.007	0.540 ± 0.008
CIFAR-100	KNN	0.749 ± 0.006	0.653 ± 0.011	0.626 ± 0.008	0.605 ± 0.006	0.612 ± 0.005

**Table 16:** Seed-42 optimization-budget sensitivity, with both accuracy columns remeasured: clean accuracy on the full 10,000-image test set and PGD-20 robust accuracy on the 2000 held-out indices through the test transform. Energy AUROC uses the full ID and CIFAR-100 test sets.

Configuration	Epochs	Clean Accuracy (10,000)	PGD-20 Accuracy (2000)	Near-OOD Energy AUROC
PGD-3	10	0.580	0.281	0.661
PGD-10	10	0.492	0.327	0.617
PGD-3	20	0.699	0.316	0.726
PGD-10	20	0.595	0.371	0.668
PGD-3	30	0.745	0.323	0.740
PGD-10	30	0.646	0.390	0.686

**Table 17:** Descriptive regression of detection AUROC on clean accuracy across the 31 models of both training studies, with the training-PGD step count added as a second term. “Steps effect” is the fitted AUROC change across the whole 0-to-10 step range. These are associations within this model pool, not causal estimates, and the two studies differ in architecture stem and Mahalanobis conditioning, as described above.

Detector	OOD Set	Slope	$R^{2}$ (Clean Accuracy)	$R^{2}$ (+ Steps)	Steps Effect	*F* (Steps)
MSP	CIFAR-100	0.560	0.995	0.997	−0.011	22.02
Energy	CIFAR-100	0.589	0.991	0.993	−0.011	7.31
KNN	CIFAR-100	0.547	0.981	0.982	+0.004	0.36
Mahalanobis	CIFAR-100	0.033	0.073	0.103	−0.009	0.93
MSP	SVHN	0.539	0.891	0.893	+0.010	0.48
Energy	SVHN	0.809	0.924	0.933	+0.032	3.41
KNN	SVHN	1.009	0.877	0.877	+0.004	0.02
Mahalanobis	SVHN	−0.042	0.004	0.013	−0.025	0.25

**Table 18:** Archived processing cost per image on Jetson AGX Orin (JetPack R36.5, MAXN, jetson_clocks, 224×224 input), with the original M4 Pro comparison. Batched cost is total batch time divided by batch size, not end-to-end latency. Separate component and full-monitor rows come from their respective timing loops.

Component	b = 1 (ms)	b = 8 (ms)	b = 64 (ms)	M4 Pro b = 1 (ms)
ResNet50 forward (MSP + Energy)	12.74	2.95	2.54	5.05
+Mahalanobis distance	13.11	2.97	2.55	5.06
+Fisher fusion (ECDF, 3 constituents)	13.84	3.02	2.55	—
CLIP ViT-B/32 zero-shot	11.78	4.06	3.25	4.42
Full monitor (ResNet50 + Mahalanobis)	13.45	2.98	2.55	—

## Data Availability

The accompanying submission supplements provide frame lists, score tables, cached reviewer-control image embeddings, and analysis scripts. File S2 supports the original driving ranking tables; camera–LiDAR results require the restricted paired scores in File S4, and night subgroup reconstruction also requires the image-statistic assignments provided in File S6. File S6 contains the completed control outputs with explicit provenance and verification limits; the full selected negative-label bank is not retained. File S7 retains historical synthetic/AT outputs; File S9 identifies and replaces the superseded ResNet comparisons and supplies the training follow-ups. Raw benchmark images and trained checkpoints are not redistributed in this package. Raw PUG, CIFAR, SVHN, BDD100K, and Boreas data remain subject to their source licenses and access procedures. Cached-score verification does not require raw images or GPU inference; full acquisition reruns require the documented data, weights, and environment.

## Supplementary Materials

The following supporting information can be downloaded at https://www.mdpi.com/article/10.3390/s26206432/s1. File S1: Original driving frame lists and hashes. File S2: Original per-frame detector scores. File S3: Sensor-chain aggregate results and archived per-sample score arrays. File S4: Camera–LiDAR synchronization, paired scores and error summaries. File S5: Embedded timings, platform record, and thermal trace. File S6: Completed reviewer-control result snapshots (E1, E2, E3, E5b, E6, E7, and E8), cached CLIP embeddings/frame IDs, acquisition-source snapshots, separately verified descriptive tables, bootstrap intervals for the independent PUG readout cells, and the unwhitened Euclidean control values for all sixteen text-free cells. File S7: Historical PUG checkpoint, corruption and adversarial-training results, and selected score arrays, with superseded ResNet comparisons explicitly identified. File S8: Separate-calibration Boreas scores, fixed-direction PUG version audit, source hashes, and an independent score-only reproducer. File S9: Preprocessing A/B audits for the original CIFAR checkpoints and CIFAR-10-C, completed five-seed training and one-seed budget-sweep results, source scripts, and checkpoint hashes. File S10: Follow-up Boreas metadata audit, official test-split/reference-pose documentation, and limits of the local archive search. Package-level scripts recompute the text-free readouts from cached embeddings and document the scope of each check.

## Appendix A. Per-Corruption Results

The complete corruption matrix is retained to show the heterogeneity behind the severity means. These point estimates use the same 2000 clean/corrupted image indices and fixed score direction. Table A1 gives the per-corruption values.

**Table A1:** Per-corruption AUROC after the ResNet input correction. C: unchanged CLIP maximum cosine; M: training-normalized ResNet18 Mahalanobis. Original raw-input values and the A/B acquisition code are archived in File S9.

Corruption	C (sev.1)	M (sev.1)	C (sev.3)	M (sev.3)	C (sev.5)	M (sev.5)
gaussian noise	0.430	0.485	0.550	0.577	0.623	0.635
shot noise	0.431	0.473	0.519	0.541	0.627	0.622
impulse noise	0.438	0.508	0.635	0.605	0.843	0.805
defocus blur	0.466	0.489	0.400	0.528	0.485	0.726
glass blur	0.691	0.533	0.700	0.508	0.783	0.516
motion blur	0.435	0.525	0.431	0.638	0.441	0.677
zoom blur	0.437	0.536	0.431	0.598	0.469	0.671
snow	0.581	0.508	0.637	0.529	0.652	0.547
frost	0.491	0.448	0.560	0.468	0.595	0.505
fog	0.447	0.468	0.396	0.445	0.504	0.558
brightness	0.495	0.497	0.492	0.492	0.532	0.480
contrast	0.433	0.458	0.355	0.501	0.498	0.912
elastic transform	0.443	0.483	0.414	0.490	0.623	0.482
pixelate	0.532	0.485	0.559	0.514	0.647	0.753
jpeg compression	0.661	0.459	0.759	0.464	0.829	0.464
